# Structural Analysis and Classification of Low-Molecular-Weight Hyaluronic Acid by Near-Infrared Spectroscopy: A Comparison between Traditional Machine Learning and Deep Learning

**DOI:** 10.3390/molecules28020809

**Published:** 2023-01-13

**Authors:** Weilu Tian, Lixuan Zang, Lei Nie, Lian Li, Liang Zhong, Xueping Guo, Siling Huang, Hengchang Zang

**Affiliations:** 1 NMPA Key Laboratory for Technology Research and Evaluation of Drug Products, School of Pharmaceutical Sciences, Shandong University, Jinan 250012, China; 2 National Glycoengineering Research Center, Shandong University, Jinan 250012, China; 3 Key Laboratory of Chemical Biology (Ministry of Education), Shandong University, Jinan 250012, China; 4 Bloomage Biotechnology Corporation Limited, Jinan 250012, China

**Keywords:** hyaluronic acid, low-molecular-weight hyaluronic acid, near-infrared spectroscopy, nuclear magnetic resonance, hydrogen bond, chemometrics, classification, machine learning, neural networks, deep learning

## Abstract

Confusing low-molecular-weight hyaluronic acid (LMWHA) from acid degradation and enzymatic hydrolysis (named LMWHA–A and LMWHA–E, respectively) will lead to health hazards and commercial risks. The purpose of this work is to analyze the structural differences between LMWHA–A and LMWHA–E, and then achieve a fast and accurate classification based on near-infrared (NIR) spectroscopy and machine learning. First, we combined nuclear magnetic resonance (NMR), Fourier transform infrared (FTIR) spectroscopy, two-dimensional correlated NIR spectroscopy (2DCOS), and aquaphotomics to analyze the structural differences between LMWHA–A and LMWHA–E. Second, we compared the dimensionality reduction methods including principal component analysis (PCA), kernel PCA (KPCA), and t-distributed stochastic neighbor embedding (t-SNE). Finally, the differences in classification effect of traditional machine learning methods including partial least squares–discriminant analysis (PLS-DA), support vector classification (SVC), and random forest (RF) as well as deep learning methods including one-dimensional convolutional neural network (1D-CNN) and long short-term memory (LSTM) were compared. The results showed that genetic algorithm (GA)–SVC and RF were the best performers in traditional machine learning, but their highest accuracy in the test dataset was 90%, while the accuracy of 1D-CNN and LSTM models in the training dataset and test dataset classification was 100%. The results of this study show that compared with traditional machine learning, the deep learning models were better for the classification of LMWHA–A and LMWHA–E. Our research provides a new methodological reference for the rapid and accurate classification of biological macromolecules.

## 1. Introduction

Hyaluronic acid (HA) is a glycosaminoglycan composed of the basic structure of disaccharides (D-glucuronic acid and N-acetylglucosamine) [1]. Due to its unique molecular structure as well as physical and chemical properties, it has physiological functions such as lubrication, moisturizing, and viscoelasticity, which makes it widely used in biomedical and clinical fields [2,3,4,5]. In 2021, HA was approved by the National Health Commission of the People’s Republic of China for use in general food in the Chinese market. HA with a molecular weight above 10^6^ Da is called high-molecular-weight HA (HMWHA), and HA with a molecular weight below 10^6^ Da is called low-molecular-weight HA (LMWHA) [6]. Compared with HMWHA, LMWHA has higher permeability and higher biological activity, such as promoting wound healing, inhibiting tumor proliferation, immune regulation, etc. [7,8,9,10], giving it broad application prospects in the fields of medicine, food, and healthcare, etc. In industry, LMWHA is usually obtained by acid degradation or enzymatic hydrolysis [11,12]. The acid degradation method has a high reaction and low cost, but it has the risk of destroying the basic disaccharide structural unit of HA, and there is residue, which poses a threat to human health [12]. The enzymatic hydrolysis has mild action and specific cleavage sites, which will not destroy the basic structure of HA [13]. Meanwhile, hyaluronidase as a natural ingredient is harmless to the human body [14], but a disadvantage is that the cost is high. In order to save production costs, some illegal producers may use acid-degraded LMWHA (LMWHA–A) in place of enzyme-hydrolyzed LMWHA (LMWHA–E), which will put the health of LMWHA users at high risk all the time. Especially when LMWHA–A is used in clinical medicine, the hidden dangers and losses caused by it are immeasurable.

For LMWHAs with the same molecular weight but different degradation methods, it is difficult to distinguish them using simple methods, whether they are solid powders or in aqueous solutions. The most effective method in the past was to use nuclear magnetic resonance (NMR) to analyze the difference in chemical structure, but the analysis of the NMR spectrum is not easy, and the test time is long, which is not conducive to continuous monitoring, and the test cost is relatively expensive. Therefore, it is necessary to establish a fast, accurate, and efficient method to distinguish LMWHAs.

The wavelength of the near-infrared (NIR) spectrum is between 780 nm and 2526 nm, and NIR can obtain molecular vibration information of hydrogen-containing groups, so it is widely used in the field of bioscience and related subjects [15,16]. NIR spectra have the characteristics of severe overlapping of spectral bands, so the spectra must be further analyzed with the help of knowledge and techniques in the field of chemometrics [17,18]. In recent years, with the rapid development of machine learning, especially deep learning, the content of chemometrics has been greatly enriched, and it is useful in solving complex problems [19].

Classification algorithms are an important branch of machine learning. Traditional machine learning classification methods include partial least squares–discriminant analysis (PLS-DA) [20], decision trees (DTs) [21], random forest (RF) [22], Naive Bayes [23], the k-nearest neighbor algorithm (KNN) [24], and support vector machines (SVMs) [25]. A traditional machine learning model belongs to shallow learning in essence. Its model has lower complexity, stronger interpretability of features, lower requirements on computer performance, and faster training speed [26]. However, its limitation lies in its limited ability to represent complex functions in the case of limited samples and computing units [27]. For complex classification problems, its generalization ability is restricted to some extent. At present, it is popular to choose the evolutionary algorithm represented by the genetic algorithm (GA) or the optimization model of the swarm intelligence algorithm represented by the particle swarm optimization (PSO) algorithm to optimize the model [28,29]. As an emerging algorithm in the field of machine learning, deep learning integrates feature learning and model building into one model by selecting different kernels and adjusting parameters through end-to-end optimization, and is proving to have good processing capability for complex non-linearly separable data [30,31]. The current popular deep learning classification methods are mainly based on convolutional neural networks (CNNs) [32], recurrent neural networks (RNNs) [33], and deep neural networks (DNNs) [34].

**Figure 1:**
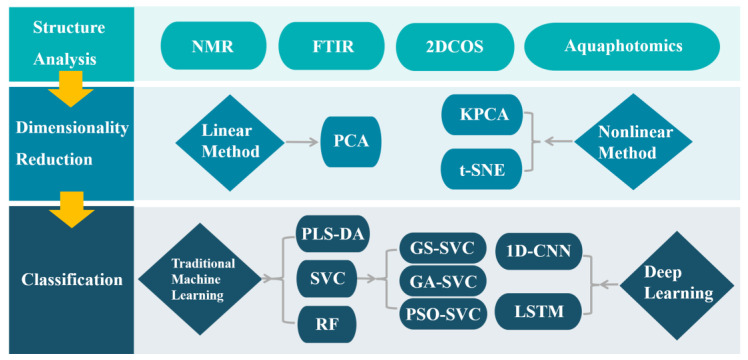
A flow diagram of this study.

In the present work, we first performed the structural analysis of LMWHA–A and LMWHA–E by NMR and two-dimensional correlated NIR spectroscopy (2DCOS). Second, the differences in the types of water molecules in the two aqueous LMWHA solutions were analyzed by applying the theory of aquaphotomics in order to explain the structural differences between the two from the side. Third, we employed a series of linear and nonlinear dimensionality reduction methods including principal component analysis (PCA), kernel PCA (KPCA), and t-distributed stochastic neighbor embedding (t-SNE) to observe the distribution of the dataset in 3D space. Fourth, we compared several traditional machine learning classification methods, and applied several intelligent optimization algorithms to improve support vector classification (SVC). Finally, we established the classification models using a one-dimensional CNN (1D-CNN) and long short-term memory (LSTM) model in deep learning. Figure 1 shows a flow diagram of this study.

## 2. Results and Discussion

### 2.1. NMR and FTIR Spectrum Description

Figure S1 shows the NMR spectra of LMWHA–A and LMWHA–E. Comprehensively analyzing the 1D and 2D spectra, it can be found that whether it was LMWHA–A or LMWHA–E, the low-field region of the carbon spectrum (chemical shifts between 168 ppm and 174 ppm) had carbon signals of carboxyl and amide groups. However, LMWHA–A has an absorption peak between 172 ppm and 173 ppm, which represented the signal obtained by the hydrolysis of amide groups. Figure S2 shows the Fourier transform infrared (FTIR) spectra of LMWHA–A and LMWHA–E solutions. The yellow area in Figure S2 highlighted the differences in band intensity, shape, and chemical shift between LMWHA–A and LMWHA–E. Among them, the difference in absorption peak from 1250 cm^−1^ to 1580 cm^−1^ can be attributed to the changes in amide group and the symmetric C-O stretching vibrations of the ether bond [35], while the difference in absorption peak between 1750 cm^−1^ and 2400 cm^−1^ can be attributed to C=O stretching and C-H bending of amide group [36]. Therefore, it can be speculated that under acid degradation conditions, the C-N bond of the amide group was cleaved, while the carboxyl group in the primary structure of HA remained. However, under enzymatic hydrolysis conditions, neither the carboxyl group nor the amide group was cleaved. On the other hand, it can be seen from the terminal carbon signal of NMR spectra that the chemical shift of the terminal carbon of the monosaccharide on the sugar chain was around 107 ppm, and the chemical shift of the terminal carbon signal of the monosaccharide generally appeared around 100 ppm [36,37]. Under acid degradation conditions, no signal was found around 109 ppm, while the signal was more abundant around 100 ppm, while in the LMWHA–E spectra, a carbon signal appeared around 107 ppm, which was the signal of the terminal carbon of the unbroken disaccharide unit. From this, it can be inferred that under acid degradation conditions, the ether bonds connecting the monosaccharides of HA are broken, while enzymatic hydrolysis does not break the ether bonds [38].

The results in this section suggest that enzymatic hydrolysis did not destroy the basic building blocks of HA, whereas acid degradation did the opposite. Figure S3 shows the deduced chemical structures of LMWHA–A and LMWHA–E. Importantly, human hyaluronidase is only capable of specific degradation of structurally intact HA [39]. Therefore, residues of LMWHA–A are at risk of accumulation in humans.

### 2.2. NIR Spectrum Description

**Figure 2:**
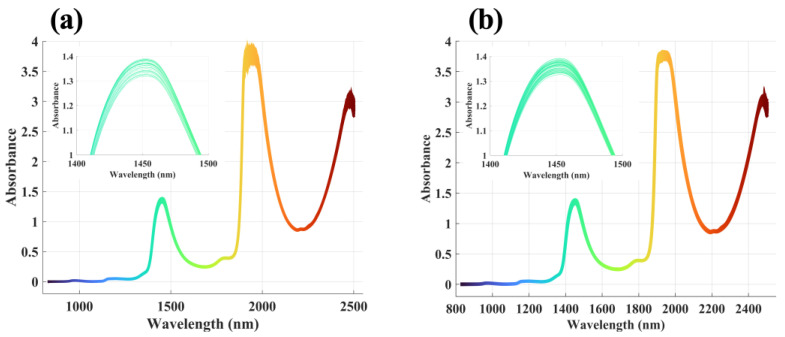
The NIR spectra of all LMWHA solution samples in the wavenumber range of 780 nm to 2500 nm. (**a**) Raw spectra; (**b**) Spectra after preprocessing with SG smoothing filter and MSC.

The raw NIR spectra of LMWHA solution samples in the 780 nm–2500 nm frequency regions are shown in Figure 2a. After preprocessing with the Savitzky–Golay (SG) smoothing filter and multiplicative Scatter Correction (MSC) method, the noise was suppressed, and the spectrum appeared smoother than the raw spectrum (as shown in Figure 2b). The whole spectrum showed the remarkable features of the water system: there were four bands around 970 nm, 1190 nm, 1450 nm, and 1940 nm, which reflected the second overtone of the OH stretching band, a combination of the first overtone of the OH stretching and OH bending band, the first overtone of the OH stretching band, and a combination of the OH stretching and OH bending band, respectively [40,41].

### 2.3. Analysis of 2DCOS Synchronous and Asynchronous Spectra

As an auxiliary analytical tool for one-dimensional spectroscopy, 2DCOS can help identify the chemical information of overlapping peaks and small peaks. In a HA solution, hydrogen bonds are formed between water molecules, between HA, and between HA and water molecules [42]. In order to facilitate the analysis of the differences between LMWHA–A and LMWHA–E in an aqueous solution, the first overtone (1300 nm–1600 nm) of O–H and hydrogen bonds of water molecules was taken as the signal region of interest, and 2DCOS synchronous and asynchronous spectra were obtained according to the Noda algorithm [43].

**Figure 3:**
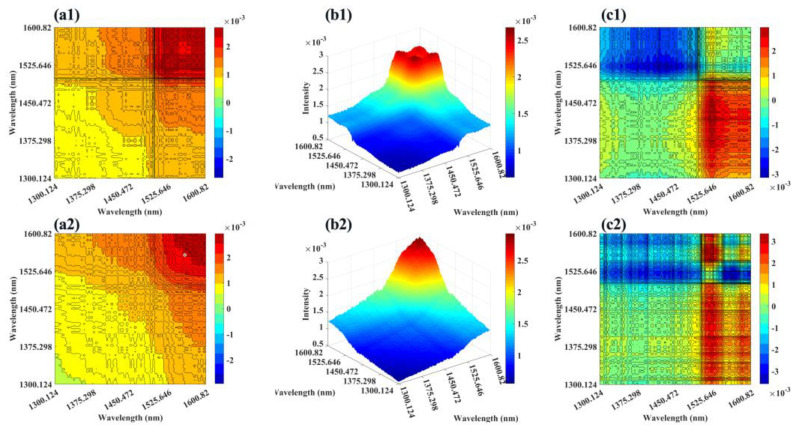
Synchronous spectra of (**a1**) LMWHA–A and (**a2**) LMWHA–E; Auto-peak spectra of (**b1**) LMWHA–A and (**b2**) LMWHA–E; Asynchronous spectra of (**c1**) LMWHA–A and (**c2**) LMWHA–E.

By comparing Figure 3a1 and Figure 3a2, it can be found that the synchronous cross-peaks of both LMWHA–A and LMWHA–E were positive at 1300 nm–1600 nm, and the peak intensity increased with the increase of wavelength; At 1500 nm–1600 nm (the first overtone stretching vibration of the hydrogen-bonded hydroxyl group), the intensity of the cross-peaks was significantly higher than that of the remaining regions reflecting the first overtone stretching vibration information of the free hydroxyl group. This indicated that acid degradation and enzymatic hydrolysis strongly disturbed the hydrogen bond in an aqueous solution.

By comparing Figure 3b1 and Figure 3b2, it can be found that LMWHA–A had four automatic peaks of similar intensity in the red region of 1525 nm–1600 nm, while LMWHA–E has only one automatic peak of maximum intensity in this region. This indicated from the side that acid degradation and enzymatic hydrolysis had different changes in HA structure.

By comparing Figure 3c1 and Figure 3c2, it can be further found that the cross-peak symbols of the asynchronous spectrum of LMWHA–A were almost the same in the range of 1550 nm to 1600 nm, while there were obvious differences in LMWHA–E. According to Noda’s theory, in the synchronous and asynchronous 2DCOS plots, the symbols of the cross-peaks located at λ_1_ and λ_2_ can be used to reveal the order of spectral intensity change at λ_1_ and λ_2_ [44]. If the same sign is observed in the synchronous and asynchronous cross-peaks, it indicates that the intensity change of λ_1_ occurs before that of λ_2_, while the opposite sign in the synchronous and asynchronous cross-peaks indicates that the band intensity change of λ_2_ occurs before that of λ_1_. Therefore, it was not difficult to determine that the disturbance sequence of enzymatic hydrolysis to the first overtone stretching vibration of the hydrogen-bonded hydroxyl group occurred before the disturbance of the first overtone stretching vibration of the free hydroxyl group. Although acid degradation has a similar tendency, the effect is not as obvious as that of enzymatic hydrolysis.

Therefore, it can be inferred that enzymatic hydrolysis can evolve HA solution in a simpler direction, and its influence on the hydrogen bond layout in the solution is obvious. However, the treatment of acid degradation is messy and has no strong directionality to the hydrogen bond layout in an aqueous solution. This is confirmed by the enzymatic digestion method retaining the primary structure of HA; that is, more hydrogen bonds tended to be formed in the solution due to the formation of more short-chain polysaccharides with complete structures. This result confirmed the inference described in Section 2.1.

### 2.4. Aquaphotomics Analysis

**Figure 4:**
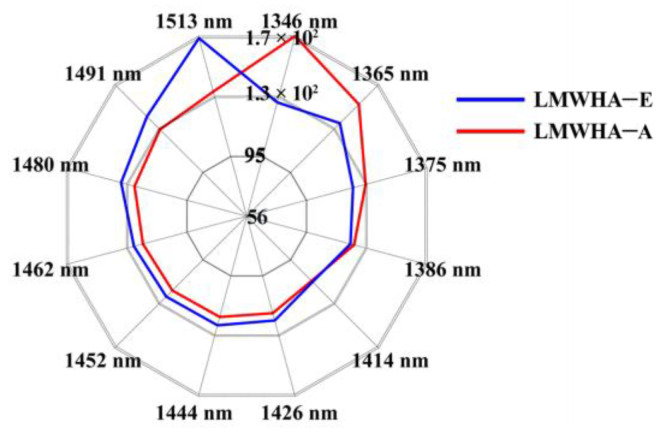
Aquagrams of LMWHA–A and LMWHA–E solutions tested at 25 °C.

The water spectrum contains information about covalent hydroxides and hydrogen bonds and is highly influenced by other molecules and environmental factors in a solution. It can be found from Figure 4 that LMWHA–A had a dominant absorption at 1346 nm–1375 nm, while LMWHA–E has a dominant absorption at 1480 nm–1513 nm. Table S1 lists the water matrix coordinates (WAMACs) and the vibrational information of the molecular structures they represent [45]. According to the analysis of Table S1, it can be found that there were more H_2_O asymmetric stretching vibrations and water solvation shells in the LMWHA–A solution, while there were more water molecules with three or four hydrogen bonds, H_2_O bending vibrations, and strongly bound water in LMWHA–E solution. This showed that LWMHA-E can more strongly associate water molecules through hydrogen bonds and promote the formation of more hydrogen bonds in aqueous solutions. This finding supported the conclusion in Section 2.3.

### 2.5. Sample Exploration by PCA, KPCA, and t-SNE

PCA is a commonly used data analysis method. It transforms the original data into a set of linearly independent representations of each dimension through linear transformation [46]. It can be used to extract the main feature components of the data and is often used for the dimensionality reduction of high-dimensional data.

**Figure 5:**
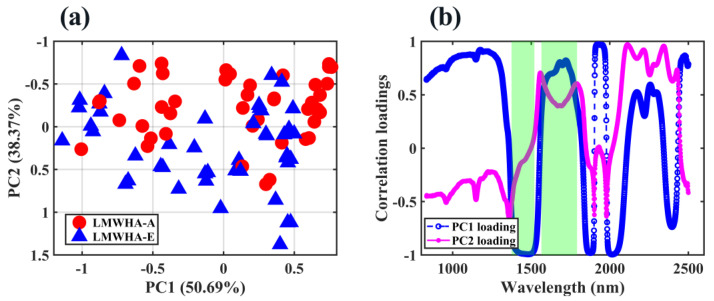
(**a**) 2D score plot and (**b**) correlation loadings plot of PC1 and PC2 of LMWHAs.

Figure 5a shows the distribution of scores of LMWHA–A and LMWHA–E in the 2D space composed of the first two principal components (PC1 and PC2). It can be found that the two types of samples were not clearly distinguished in the spatial distribution. Figure 5b shows the correlation loadings of PC1 and PC2. It is not difficult to find that in the wavelength range covered by the green area, the direction of change of the correlation loadings of PC1 and PC2 was different (it was also different at the end of the entire wavelength, but as shown in Figure 2, the absorbance values at those wavelengths were too high, so that we did not care about these variables). Coincidentally, the green area highly overlaps with the first overtone of O–H and hydrogen bonds of water molecules, which suggested that changes in water molecules and hydrogen bonds were important internal factors for the distinction between LMWHA–A and LMWHA–E.

A PCA can identify underlying dominant features and provide a more concise and straightforward summary of relevant covariates, but it can only be applied to linearly separable datasets. If we apply a PCA to a non-linear dataset, we may obtain a poor dimensionality reduction result. LMWHA–A and LMWHA–E have a high similarity in structure, so it is necessary to try nonlinear dimensionality reduction methods. KPCA uses a kernel function to map the dataset to a high-dimensional feature space (a reproducing kernel Hilbert space), and then performs PCA in this high-dimensional space to achieve nonlinear dimensionality reduction of the data [47,48].

**Figure 6:**
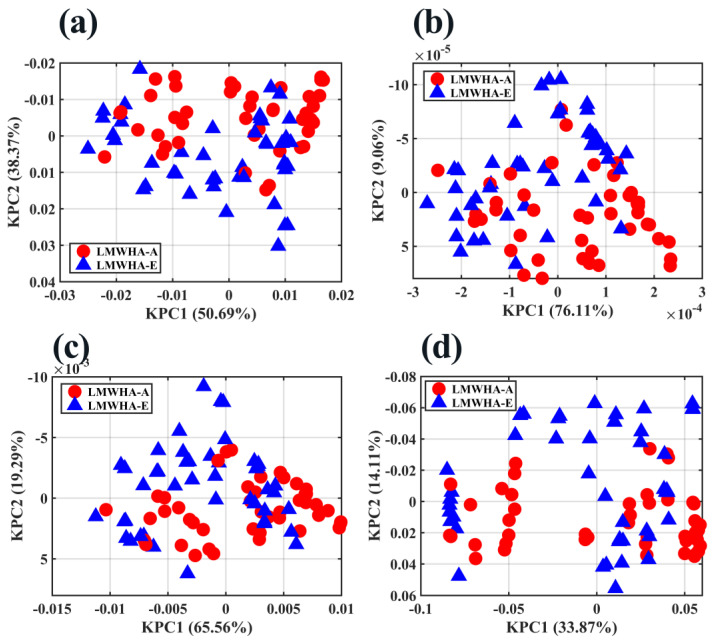
2D score plots of KPCA with (**a**) Gaussian kernel, (**b**) polynomial kernel, (**c**) sigmoid kernel, and (**d**) Laplacian kernel.

As shown in Figure 6, multiple kinds of kernel functions (Gaussian, polynomial, sigmoid, and Laplacian) were used for dimensionality reduction and visualization. From the 2D score plots, no matter which kernel function was used, LMWHA–A and LMWHA–E were not effectively distinguished. Although PCA and KPCA are mainly used for dimension reduction rather than cluster analysis or visualization, the results of these two methods at least illustrate one fact: LMWHA–A and LMWHA–E share many similarities in structure, resulting in high similarity in many features of their NIR spectra.

T-SNE is another popular method for nonlinear data dimensionality reduction, which tries to keep similar instances adjacent and separate dissimilar instances while reducing dimensionality [49]. One of its main advantages is that the original features of the dataset are preserved as much as possible in the mapping from high-dimensional to low-dimensional space; that is, two data points that are similar in high-dimensional space are also similar when mapped to low-dimensional space [26]. T-SNE is widely used in visualization in fields such as bioinformatics, biomedical signal processing, and natural language processing.

**Figure 7:**
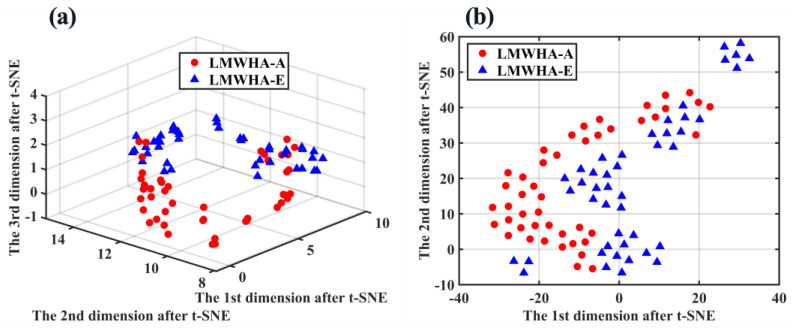
(**a**) Distribution map of LMWHA–A and LWMHA-E in 3D space after t-SNE dimensionality reduction; (**b**) Distribution of LMWHA–A and LWMHA-E in 2D space after t-SNE dimensionality reduction.

Figure 7a,b shows the visualization results of the t-SNE algorithm reducing the data dimension to 3D space and 2D plane, respectively. It can be seen that LMWHA–A and LMWHA–E were well distinguished in 3D space. Although there were still a small number of samples mixed together, the overall visualization is better than PCA and KPCA. Considering the principle of t-SNE, we believe that this is because the t-SNE algorithm introduces t-distribution, which is a kind of long-tail distribution, which can tolerate the influence of outliers on most samples to a higher degree, so as to make better use of the overall characteristics of data and improve the robustness of the algorithm.

### 2.6. Sample Classification Based on Traditional Machine Learning Methods

#### 2.6.1. PLS-DA

**Figure 8:**
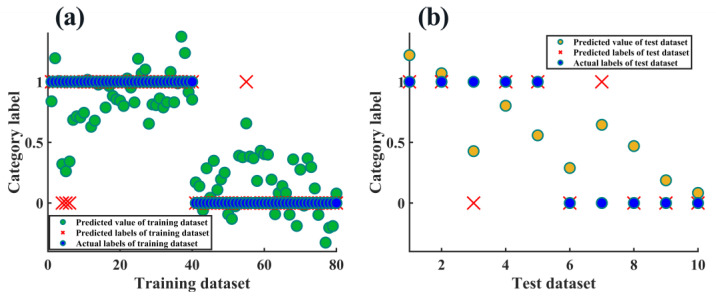
Classification results of (**a**) training dataset and (**b**) test dataset using PLS-DA method.

PLS-DA is essentially a classification method based on eigenvariables. It can decompose the spectra matrix and the response variable orthogonally at the same time, establish a regression relationship between them, and obtain a better classification effect than PCA in the projection map [20]. For the binary classification problem to be solved in this study, the response variables of the known categories were set to 0 (LMWHA–A) and 1 (LMWHA–E), and then the predicted response variable values were rounded and compared with the real labels to finally calculate the classification accuracy. As shown in Figure 8, after leave-one-out cross-validation (LOOCV), 4 of 80 training set samples were misclassified (see Figure 8a; misclassified samples are marked in red), and 2 of 10 test set samples were misclassified (see Figure 8b; misclassified samples are marked in red), and the results were not perfect.

#### 2.6.2. SVC and Optimized SVCs

**Figure 9:**
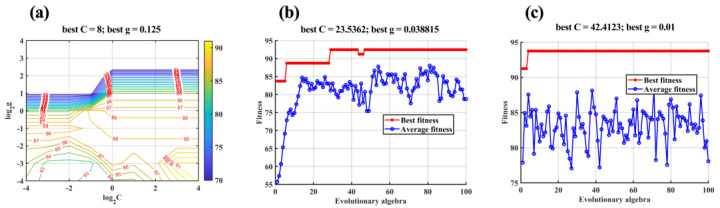
Parameter selection results of (**a**) GS−SVC, (**b**) GA−SVC, and (**c**) PSO−SVC.

The basic idea of SVC is to establish a hyperplane as a decision surface based on the principle of structural minimization, which maximizes the isolation margin between samples of different categories [25]. SVC first uses the selected kernel function to nonlinearly map the training set from the input space to a high-dimensional feature space and then completes linear classification in this space. Therefore, different kernel functions lead to different classification effects. At present, the kernel function that is recognized as having the best effect in the classification problem of small sample data is the radial basis function (RBF) [50]. However, the hyperparameters C and g in the BRF kernel function affect the performance of the classifier [51]. C is the penalty coefficient, that is, the tolerance for errors. The larger C is, the more intolerable the error, and it is easy to overfit. The smaller C is, the easier it is to underfit. If C is too large or too small, the generalization ability deteriorates. G implicitly determines the distribution of the data after it is mapped to the new feature space. The larger g is, the fewer support vectors, and the smaller g is, the more support vectors. The number of support vectors affects the speed of training and prediction. In order to improve the accuracy of a classification and speed up the operation, some optimized algorithms have been proposed. Among them, grid search (GS), GA, and PSO are the three most popular optimization methods at present. GS optimizes the model by traversing given parameter combinations and determines the best C and g through cross-validation [52]. GA is a kind of stochastic optimization search algorithm that evolved from the evolution law of biology (genetic mechanism of survival of the fittest). It can deal with multiple individuals in a group at the same time, reducing the risk of falling into a locally optimal solution [53]. PSO is a stochastic optimization algorithm based on swarm intelligence. It imitates the foraging behavior of birds and compares the search space to the flight space of birds. The optimal solution to be found is equivalent to the food that birds are looking for. Through continuous iterations and calculation of fitness value, the optimal solution is finally obtained [54]. Therefore, three intelligent algorithms, GS, GA, and PSO were used to optimize the parameters of the SVC kernel and were compared with the traditional SVC. Figure 9 shows the parameter selection results of GS−SVC, GA−SVC, and PSO−SVC.

Figure S4 shows the confusion matrix of the training dataset through SVC, GS−SVC, GA−SVC, and PSO−SVC. It can be found that the classification effect of GS−SVC on the training dataset reached 100%, which was the best among the three optimization methods. Figure S5 shows the receiver operating characteristic (ROC) curve and the area under the curve (AUC) of SVC and three optimized SVC of the training dataset. As an auxiliary evaluation index of the confusion matrix, the closer the ROC curve is to the upper left corner, the larger the AUC area and the better the performance of the classifier. The AUC of GS−SVC was 1, indicating that it had the best classification effect on the training dataset.

Figure S6 shows the confusion matrix of the test dataset with SVC and three optimized SVC. It can be found that the accuracy rate of GA−SVC for the classification of the test dataset was 90%, and the accuracy rates of GS−SVC and PSO−SVC were 80%, both of which are higher than the traditional SVC (70%). Figure S7 shows the ROC curve and AUC of the test dataset with SVC and three optimized SVC. It can be found that the AUC of GA−SVC classification was the largest, reaching 0.96, and the AUC after GS−SVC and PSO−SVC classification were both 0.8, which were higher than the AUC of the traditional SVC method (0.64).

Table 1 shows all of the classification metrics of SVC, GS−SVC, GA−SVC, and the PSO−SVC model, including accuracy, precision, specificity, sensitivity (recall), F_1_ score, and AUC. Based on the analysis of various indicators, the GS−SVC algorithm performed the best in the classification of the training dataset, and the GA−SVC algorithm performed the best in the classification of the test dataset. Compared with the traditional SVC, the three optimization methods improved the classification effect.

Nu-SVC is an SVC with a polynomial kernel, and its default degree is three. Nu represents the upper limit of the error rate of the training dataset, or the lower limit of the percentage of the support vector, which has a similar function to the penalty coefficient C in the SVC algorithm, and can control the intensity of the penalty. The value range of nu is (0,1], and the default value is 0.5. In order to fully compare the classification performance of nu-SVC, we calculated the models with nu values of 0.5, 0.6, 0.7, 0.8, and 0.9 (based on the situation in that we intend to solve a binary classification problem). Figures S8–S11 show the confusion matrix and ROC curves of the nu-SVC training dataset and test dataset.

#### 2.6.3. RF Algorithm

**Figure 10:**
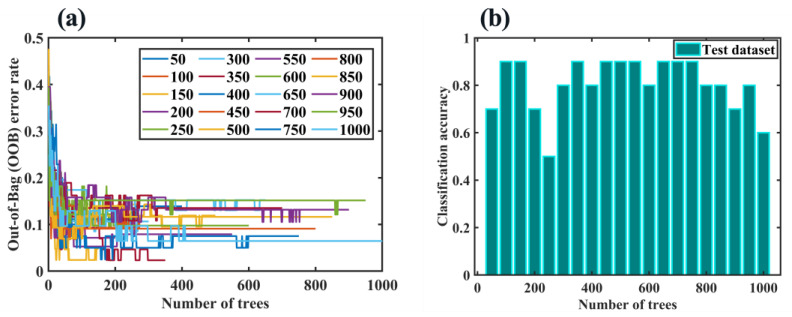
(**a**) OOB error rate and (**b**) classification accuracy with different numbers of DTs.

RF is an algorithm that integrates multiple DTs through the idea of ensemble learning [55]. Its basic unit is a DT, and each decision tree is a classifier. For an input sample, n trees have n classification results. RF integrates the classification voting results of all DTs and designates the category with the most votes as the final output. The number of DTs is a key factor affecting the classification accuracy of the RF model. Therefore, we examined the impact of 50 to 1000 DTs (with an interval of 50) on model performance. It should be pointed out that out-of-bag (OOB) error is a common index for evaluating RF fitting ability, and it tends to be stable with the increase of model iterations. The larger the stable value is, the worse the fitting ability of the model is; otherwise, the better the fitting ability is. It can be seen from Figure 10a that although all OOB error rates were lower than 0.2, the OOB error rate was not zero no matter the number of DTs, which indicated that RF’s verification effect on the training dataset is not perfect. Figure 10b shows the RF classification results of the test dataset under different DTs. It can be found that the highest classification accuracy was not 100%, but in most cases, it can reach 80% or 90%. Overall, RF achieved similar classification prediction results to optimized SVCs shown in Section 2.6.2.

An important function of RF is to calculate the importance of features. Figure S12 shows the mean decrease in accuracy and Gini index. The larger the mean decrease in accuracy and Gini index, the higher the importance of the feature. By converting the feature coordinates into wavenumber coordinates, it can be found that after RF calculation, 1300 nm–1600 nm had the highest importance in the entire wavelength range, confirming the correctness of the theoretical basis for the application of aquaphotomics to the analysis of LMWHAs.

### 2.7. Sample Classification Based on Deep Learning Methods

#### 2.7.1. 1D-CNN

A CNN is a feedforward neural network [56]. Its artificial neurons can respond to surrounding units within a part of the coverage area [57]. The weights and biases of a CNN model are tuned through backpropagation without the manual setting of parameters. It is currently a very popular deep-learning method in the field of computer vision. CNN is widely used in the processing of 2D image and 3D action signals, but it is still in its infancy in the analysis and application of 1D signals [58,59], especially NIR. Unlike the classic CNN, the moving direction of the convolution kernel of 1D-CNN is one-dimensional. Figure S13 shows the model architecture of our 1D-CNN (named 1D-CNN-7), which had seven neural layers: the input layer, convolution layer, rectified linear unit (ReLU) layer, maxpooling layer, fully connected (FC) layer, softmax layer, and output layer. Among these layers, the convolution layer extracts different features of the spectral matrix through convolution operations; the ReLU function can solve the problem of gradient explosion or gradient disappearance, and speed up the convergence process at the same time; the role of the max-pooling layer is to extract features again, and each neuron of it performs a pooling operation on the local receptive field; the fully connected layer can integrate the local information with category discrimination in the convolutional layer or the max-pooling layer; the softmax layer can normalize a numerical vector into a probability distribution vector, making the classification result more accurate [57].

**Figure 11:**
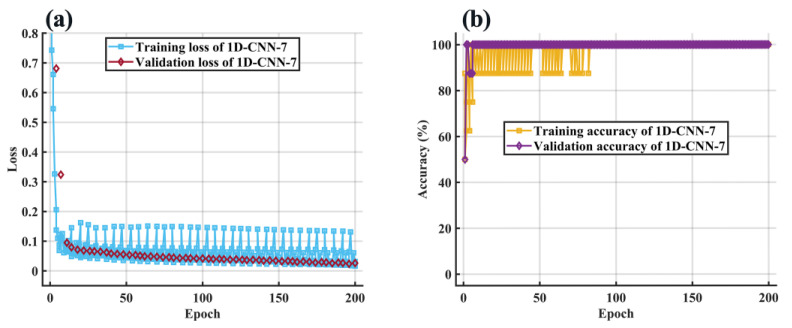
(**a**) The loss and (**b**) the accuracy curve of the 1D-CNN-7 training process.

**Figure 12:**
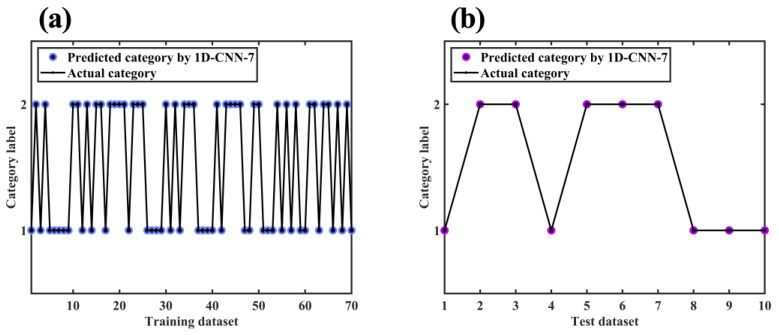
Classification results for (**a**) training dataset and (**b**) test dataset of 1D-CNN-7.

Figure 11 depicts the loss and the accuracy curve of the 1D-CNN-7 training process. It can be seen that as the number of iterations increased, the loss curves of the training set and the cross-validation set tended to fit to zero, and the accuracy tended to fit to 100%. When the number of epochs exceeded 80, the classification accuracy of the training set and cross-validation set stabilized at 100%. Figure 12 depicts classification results for the training dataset and test dataset with 1D-CNN-7. The results showed that the 1D-CNN-7 model had an excellent fitting and generalization ability.

Currently, applying 1D-CNN to the field of NIR spectroscopy is in an exploratory and popular stage. 1D-CNN combined with NIR spectroscopy has achieved satisfactory results in the fields of herbal species identification [59,60], tissue cancer detection [61], and fruit traits [62], etc. To the best of our knowledge, to date, this study is the first application of 1D-CNN combined with NIR spectroscopy for the classification issue of polysaccharides.

#### 2.7.2. LSTM

LSTM is a special and popular RNN, which is mainly used to solve the problem of gradient vanishing and gradient explosion during long sequence training [63]. Compared with other neural networks, LSTM is better at processing data with sequence changes, such as speech signals [64]. In our study, spectral data were regarded as data of sequence changes, and the LSTM model as shown in Figure S14 was constructed (the basic unit of LSTM can be seen in Figure S15 [65]). The function of the dropout layer is to add a probabilistic process to the neurons of each layer on the basis of the normal neural network to randomly discard some neurons to prevent overfitting. As far as we know, this study is the first time that LSTM and NIR spectra are combined to apply to the classification of biological macromolecules, especially polysaccharides.

**Figure 13:**
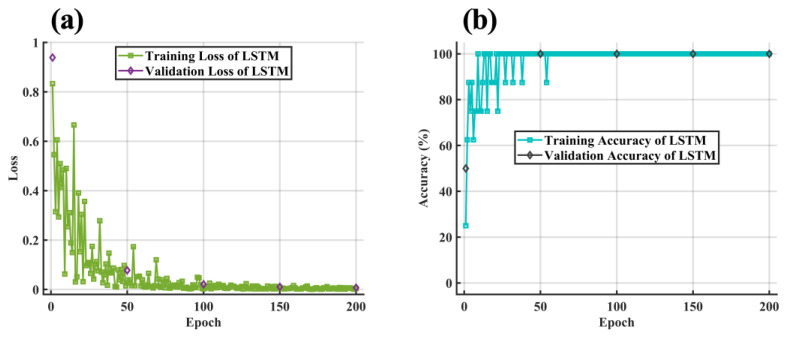
(**a**) The loss and (**b**) the accuracy curve of the LSTM training process.

**Figure 14:**
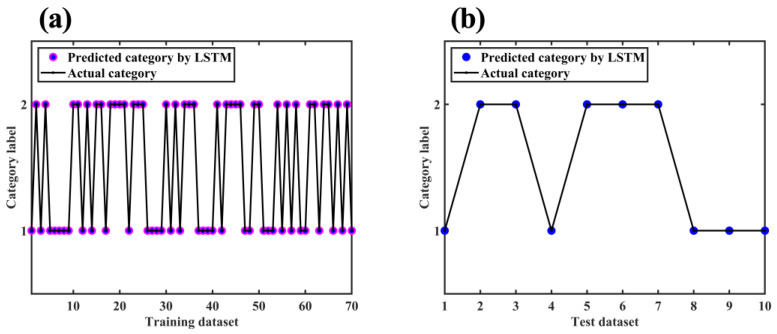
Classification results for (**a**) training dataset and (**b**) test dataset with LSTM.

Figure 13 shows the loss and the accuracy curve of the LSTM training process. Similar to the phenomenon in Section 2.7.1, with the increase in the number of iterations, the loss curve of the training set and the cross-validation set tended to zero fitting, and the accuracy tended to be 100% fitting. When the number of epochs exceeded 52, the classification accuracy of the training set and cross-validation set was stable at 100%. As shown in Figure 14, the classification accuracy of both the training dataset and test dataset after processing by the LSTM model was 100%, indicating that the effect of the LSTM algorithm was satisfactory.

Section 2.7.1 and Section 2.7.2 fully proved the superiority of the deep learning method in this research. In the past, we may have been troubled by the feature selection problem brought about by the high dimensionality of the NIR spectrum, but now deep learning happens to be able to properly deal with high-dimensional data and mine more information from it [66]. Traditional machine learning is shallow learning, and the performance of the model is highly dependent on effective feature wavelength extraction, which not only increases the complexity of the analysis work, but also heavily relies on the experience of researchers. Contrary to traditional machine learning methods, deep learning has excellent feature self-learning ability. The reason why the two deep learning models we used can achieve better results than traditional machine learning in our research, in addition to relying on the many advantages of the deep learning method itself, also needs to be attributed to the material basis of our research object—LMWHAs have complex intramolecular and intermolecular interactions in aqueous solution. We believe that in the near future, deep learning will make more dazzling breakthroughs in the application of spectral analysis methods represented by NIR spectroscopy that require the use of chemistry methods to analyze biological macromolecules, whether it is in the innovation of neural network structures or in solving more practical problems.

## 3. Materials and Methods

### 3.1. Samples

The average relative molecular weights of LMWHA–A and LMWHA–E were both 10.0 kDa. LMWHA–A and LMWHA–E were dissolved in deionized water at a concentration of 0.5 mg/mL. There were 9 batches of 90 LMWHA solutions. Each batch had 10 LMWHA solutions, of which 5 were LMWHA–A and the other 5 were LMWHA–E. All samples were in sterile packaging and stored in a 4 °C refrigerator for no more than 7 d prior to spectrum collection. Both LMWHA–A and LMWHA–E were derived from HMWHA with a molecular weight of about 2.0 × 10^6^ Da. The former needs to react for three hours at a pH value of 1.5~2.0 and a temperature of 80 °C, while the latter needs to react for three hours at a pH value of 5.5~6.0 and a temperature of 37 °C. All samples were prepared and provided by Bloomage Biotechnology Co., Ltd. (Jinan, China).

### 3.2. NMR Spectral Data Acquisition and Processing

The NMR spectra were recorded at a temperature of 25 °C using a Bruker Avance 600 spectrometer (Bruker, Billerica, MA, USA). The nuclear magnetic hydrogen spectrum (^1^H-NMR), nuclear magnetic carbon spectrum (^13^C-NMR and DEPT 135°) and two-dimensional nuclear magnetic correlation spectrum (^13^C-^1^H HSQC) were measured. The dried samples were dissolved in deuterium oxide and placed in an NMR tube with an inner diameter of 0.5 mm for testing. The test frequency for ^1^H-NMR was 600 MHz, and for ^13^C-NMR and DEPT 135° it was 150 MHz [67]. The number of sample collection points for each type of NMR is 64 K. The number of scans for ^1^H-NMR was 128, while the number of scans for other NMRs was 16. The recovery delay was 2 s. The free induction decay (FID) signal measured by the NMR instrument was imported into MestReNova 14.0.1 software (Mestrelab Research, Bajo, Santiago de Compostela, Spain) for Fourier transformation, and the NMR spectra were obtained after phase correction and baseline correction [68]. The spectra were then saved as ASCII files and imported into SpecAlign 2.4.1 software (University of Oxford, Oxford, England) for peak matching [69].

### 3.3. FTIR Spectral Data Acquisition

FTIR spectra were collected using an Alpha II FTIR spectrophotometer (Bruker, Billerica, MA, USA) with a liquid cell module. The resolution was set to 2 cm^−^^1^. The sampling temperature was set at 35 °C. Use the default number of scans of the instrument. In order to ensure the stability of the spectra, the instrument was preheated for more than 30 min.

### 3.4. NIR Spectral Data Acquisition and Sample Set Division

All NIR spectra were acquired by using a MATRIX-F FT-NIR spectrometer (Bruker, Billerica, MA, USA) equipped with a 1 mm cuvette. The spectral range is from 12,800 cm^−^^1^ to 4000 cm^−^^1^ (780 nm to 2500 nm). The resolution is 2 cm^−^^1^. The spectrometer cannot be used until it has been switched on for 30 min and has passed the self-test procedure. Taking air as a reference and subtracting its absorbance from the sample spectrum, the sample test temperature was 25 °C. The number of scans was set to 64. Each sample to be tested was divided into three equal volumes, and their spectra were collected and averaged to serve as the final spectrum of the sample. Before building the machine learning models, a total of 80 samples in the first 8 batches were divided into a training set, and a total of 10 samples in the last batch were divided into a test set. The test set samples did not participate in any model cross-validation or parameter-seeking process. The size of the original spectral matrix is 90 (number of samples) × 4148 (number of variables).

### 3.5. NIR Spectral Preprocessing

In addition to the required sample characteristics, the information collected by NIR spectroscopy is often doped with unwanted irrelevant information and noise, such as stray light, strong electrical noise, and man-made noise in the transmission process [70]. Preprocessing spectral data can reduce system noise and enhance spectral features. The SG smoothing filter is a polynomial smoothing algorithm based on the principle of least squares, which can retain useful information in the analyzed signal and eliminate random noise [71]. MSC can effectively eliminate the spectral differences caused by different scattering levels of samples, thereby enhancing the correlation between spectra and data [72]. The two algorithms described above were used to preprocess the NIR spectra.

### 3.6. 2DCOS Analysis

2DCOS is one of the tools widely used for in-depth analysis of vibrational spectral data including NIR spectra [73]. In our present study, the perturbing factors of the 2DCOS were different degradation modes [74]. The 2DCOS is composed of synchronous and asynchronous spectra on the spectrogram [43]. The synchronous correlation spectrum is obtained as a covariance matrix of the measured spectra and the asynchronous correlation spectrum as a product of the matrix of measured spectra and the Hilbert–Noda transform [43]. The synchronous correlation is symmetric about the main diagonal. The peak located on the main diagonal is called the automatic peak. The automatic peak is always a positive peak, and its intensity represents the sensitivity of the absorption peak there to external disturbances. The peaks outside the main diagonal are called cross-peaks, which can be positive or negative, and their appearance indicates that there is a synergistic response between functional groups to external perturbing factors. A positive cross-peak indicates that the peak intensities of the two functional groups increase or decrease in the same direction with the change of external disturbance, and a negative cross-peak indicates opposite changes [75]. The asynchronous correlation is antisymmetric about the main diagonal. It has no automatic peaks, only cross-peaks outside the diagonal, representing whether there is strong chemical interaction, direct connection, or pairing between functional groups [76]. The asynchronous correlation can greatly improve the resolution of the spectrum.

### 3.7. Aquaphotomics Analysis

Aquaphotomics is a novel and efficient theory for the analysis of water systems, which uses the absorption spectral features of water to characterize samples to gather information about chemical composition and environmental conditions in an indirect manner [45]. Hydrogen bond is the main factor affecting the conformation of HA in an aqueous solution [77]. Aquaphotomics is able to analyze hydrogen bond information in water systems, so this method is particularly suitable for our study. Like most of the wavelengths chosen for research in the field of aquaphotomics, the near-infrared region of the first overtone of water at 1300 nm–1600 nm was selected for analysis in this study. The WAMACs refer to a protocol of aquaphotomics analysis proposed by Prof. Tsenkova and determined by an array of analyses [78]. Then, normalization was carried out at the selected absorbance band, and the results were finally presented in the form of radar maps.

### 3.8. Data Dimensionality Reduction

Data dimensionality reduction is beneficial to eliminating a large number of redundant or irrelevant variables contained in spectral data so as to realize the description of data with less feature dimensionality, which is usually used as a preprocessing step of traditional classification algorithms [79,80]. The dimensionality reduction effects of PCA, KPCA, and t-SNE were compared.

### 3.9. Sample Classification Based on Machine Learning Methods

Compared with traditional machine learning methods, deep learning generally does not require human intervention in the feature selection or dimensionality reduction process and has advantages in high-dimensional and large-sample data processing [81,82,83]. Based on this, we compared the classification effects of traditional machine learning methods (PLS-DA, SVC, and RF) and deep learning methods (CNN and LSTM). Since the calculation results of Section 3.8 were ultimately unsatisfactory, the input matrix of any classification model did not come from data dimension reduction, that is to say, the size of the input matrix of all classification models was 90 × 4148. In this part of the work, we also nested intelligent optimization algorithms including GS, GA, and PSO for SVC. After preprocessing the spectral data and before entering the machine learning step, normalization was conducted according to formula 1 (where X represents the spectral matrix before normalization, X_max_ represents the maximum value in the matrix, X_min_ represents the minimum value in the matrix, and X’ represents the matrix after normalization), aiming to avoid the influence of outliers and extreme values. All classification models were run more than 10 times to avoid accidental errors. Confusion matrices were used to characterize the accuracy, precision, specificity, sensitivity (recall), and F_1_ score of the classification results, and these five indicators were calculated according to formulas 2–6 [84,85]. For each confusion matrix, the rows corresponded to the predicted class and the columns corresponded to the true class. The diagonal cells corresponded to observations that were correctly classified which are called true positive (TP) and true negative (TN). The off-diagonal cells corresponded to incorrectly classified observations which are called false positive (FP) and false negative (FN). Both the number of observations and the percentage of the total number of observations were shown in each cell. The column on the far right of each matrix plot showed the percentages of all the examples predicted to belong to each class that was correctly and incorrectly classified. The row at the bottom of the plot showed the percentages of all the examples belonging to each class that were correctly and incorrectly classified. The cell in the bottom right of the plot showed overall accuracy. Meanwhile, the ROC curve and the AUC were used as the evaluation indexes of the classification results [86].

(1) $$X^{\prime }=\frac{X\;-\;\mathrm{Xmin}}{\mathrm{Xmax}\;-\;\mathrm{Xmin}}$$

(2) $$\mathrm{Accuracy}\;\left(\%\right)=\frac{\mathrm{TP}+\mathrm{TN}}{\mathrm{TP}+\mathrm{FN}+\mathrm{FP}+\mathrm{TN}}\;\times \;100$$

(3) $$\mathrm{Precision}\;(\%)=\frac{\mathrm{TP}}{\mathrm{TP}+\mathrm{FP}}\;\times \;100$$

(4) $$\mathrm{Specificity}\;(\%)=\frac{\mathrm{TN}}{\mathrm{TN}+\mathrm{FP}}\;\times \;100$$

(5) $$\mathrm{Sensitivity}\;\left(\%\right)=\mathrm{Recall}\;\left(\%\right)=\frac{\mathrm{TP}}{\mathrm{TP}+\mathrm{FN}}\;\times \;100$$

(6) $$F_{1}\;\mathrm{Score}=\frac{2\;\times \;\mathrm{Precision}\;\times \;\mathrm{Recall}}{\mathrm{Precision}+\mathrm{Recall}}$$

where TP = true positive, TN = true negative, FP = false positive, and FN = false negative.

### 3.10. Programming Language

MATLAB R2022a (MathWorks Inc., Natick, MA, USA) was used for calculation and visualization.

## 4. Conclusions

The penetration ability of LMWHAs has been further improved compared with that before degradation, so it has broad application prospects in the macromolecules field all over the world. Although both acid degradation and enzymatic hydrolysis can obtain LMWHAs, the former is harmful to human health and the environment. The accurate classification of LMWHA–A and LMWHA–E is beneficial to avoid health risks caused by the accumulation of chemical reagents and free residues. In this study, NIR spectroscopy combined with machine learning methods is a proven solution that is fast, accurate, environmentally friendly, and low-cost.

NMR, FTIR, 2DCOS, and aquaphotomics were used to analyze the difference in chemical structure between LMWHA–A and LMWHA–E, which is a prerequisite for accurate classification. In order to intuitively understand the spatial distribution of the two types of samples and eliminate the multicollinearity of the data, the applicability of linear (PCA) and nonlinear (KPCA and t-SNE) methods to the NIR spectra is compared. Then, based on the NIR spectra of samples, some representative machine learning methods were used to classify and identify LMWHA–A and LMWHA–E solutions. However, traditional machine learning methods (PLS-DA, SVC, and RF) did not perform adequately in classification. Finally, we tested the 1D-CNN-7 and LSTM models in the deep learning method and found that both models had excellent classification results.

It is worth mentioning that in order to improve model performance, the traditional NIR analysis method needs manual feature selection, while deep learning enables the computer to automatically learn the pattern features, which reduces the workload and has advantages in the study of complex systems due to its strong function approximation ability. In summary, we successfully classified two LMWHA solutions quickly and accurately based on NIR spectroscopy and deep learning. At the same time, our research is the first practice of comparing traditional machine learning and deep learning in the LMWHAs classification, which provides a methodological reference for the classification of biological macromolecules, especially polysaccharides.

## Figures and Tables

**Table 1:** Classification metrics of SVC, GS−SVC, GA−SVC, and PSO−SVC models.

Method	Accuracy (%)	Precision (%)	Specificity (%)	Sensitivity/ Recall (%)	F_1_ Score	AUC
Training of SVC	90	92.5	92.1	88.1	90.2	0.9706
Training of GS−SVC	100	100	100	100	100	1
Training of GA−SVC	98.8	97.5	97.6	100	98.7	0.9819
Training of PSO−SVC	93.8	95	94.9	92.7	93.8	0.9750
Test of SVC	70	60	66.3	75	66.7	0.6400
Test of GS−SVC	80	80	80	80	80	0.8000
Test of GA−SVC	90	100	100	83.3	90.9	0.9600
Test of PSO−SVC	80	60	71.4	100	75	0.8000

## Data Availability

The data presented in this work are available in the article and supplementary materials.

## Supplementary Materials

The following supporting information can be downloaded at: https://www.mdpi.com/article/10.3390/molecules28020809/s1, Figure S1: NMR spectra; Figure S2: Confusion matrices; Figure S3: ROC curves; Figure S4: Confusion matrices; Figure S5: ROC curves; Figure S6: Mean decrease in accuracy and Gini index by RF; Figure S7: LSTM unit; Figure S8: Confusion matrices; Figure S9: ROC curves; Figure S10: Confusion matrices; Figure S11: ROC curves; Figure S12: Mean decrease in accuracy and Gini index by RF; Figure S13: Model architecture; Figure S14: Model architecture; Figure S15: Basic unit; Table S1: WAMACs.
