# Supplementary material for: Structural Analysis and Classification of Low-Molecular-Weight Hyaluronic Acid by Near-Infrared Spectroscopy: A Comparison between Traditional Machine Learning and Deep Learning
Source: Molecules. 2023 Jan 13;28(2):809. doi: 10.3390/molecules28020809 (PMC9862636; doi:10.3390/molecules28020809)
Supplement: Supplementary file 1 [file molecules-28-00809-s001.zip › molecules-2131713-supplementary.pdf]

Supplementary Figures

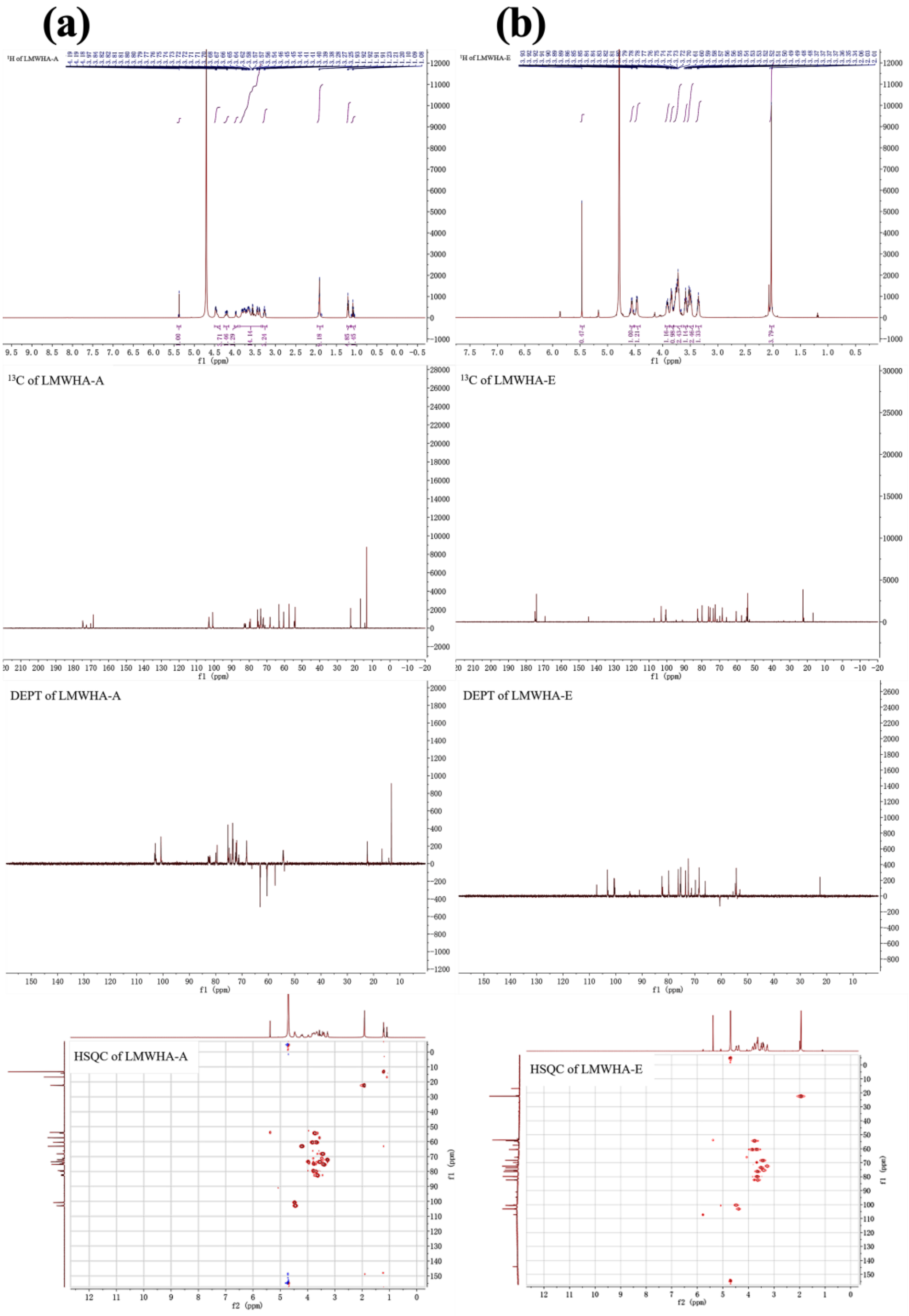

Figure S1. The ^{1}H-NMR, ^{13}C-NMR, DEPT 135°, and ^{13}C-^{1}H HSQC spectra of (**a**) LMWHA-A and (**b**) LMWHA-E.

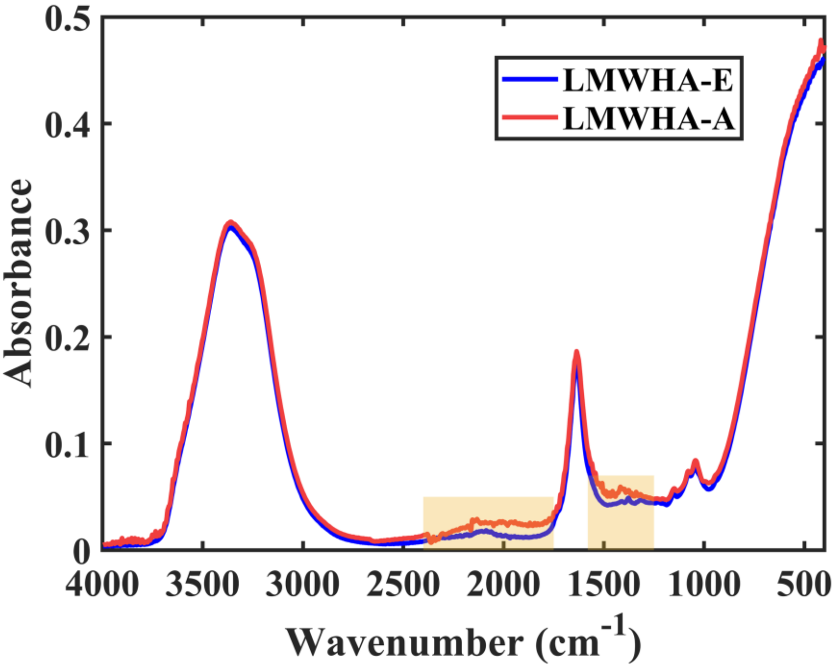

Figure S2. The FTIR spectra of LMWHA-A and LMWHA-E solutions.

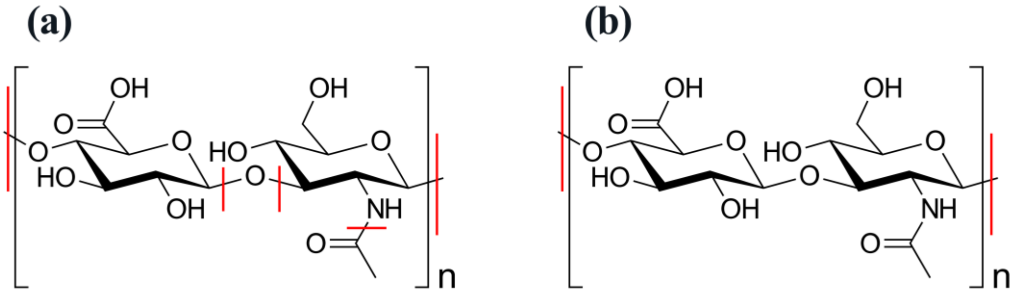

Figure S3. Deduced chemical structures of (**a**) LMWHA-A and (**b**) LMWHA-E (red indicates site of action).

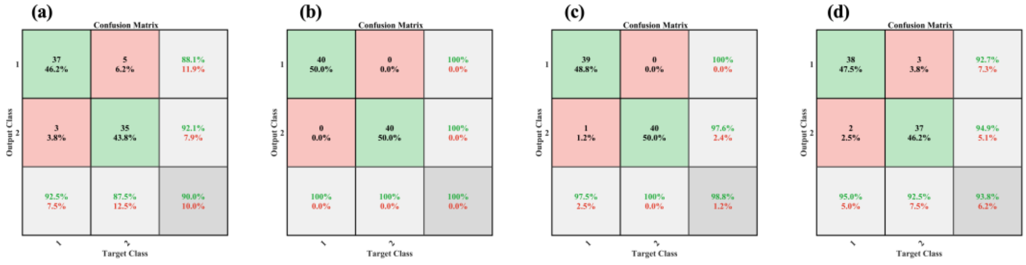

Figure S4. Confusion matrix of training dataset with (**a**) SVC, (**b**) GS-SVC, (**c**) GA-SVC, and (**d**) PSO-SVC.

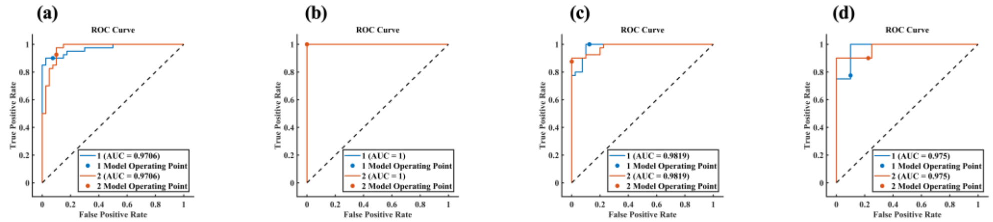

Figure S5. ROC curve of training dataset with (**a**) SVC, (**b**) GS-SVC, (**c**) GA-SVC, and (**d**) PSO-SVC.

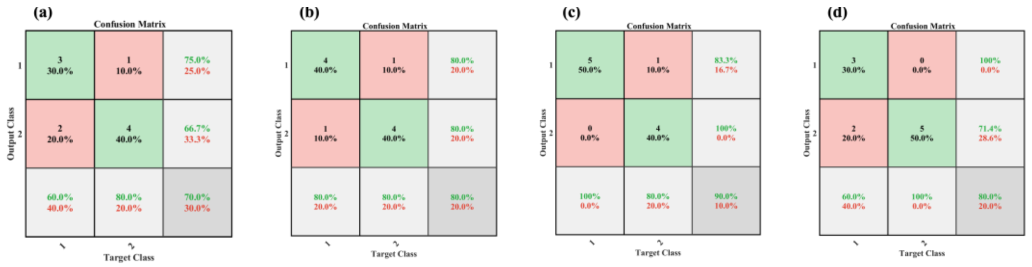

Figure S6. Confusion matrix of test dataset with (**a**) SVC, (**b**) GS-SVC, (**c**) GA-SVC, and (**d**) PSO-SVC.

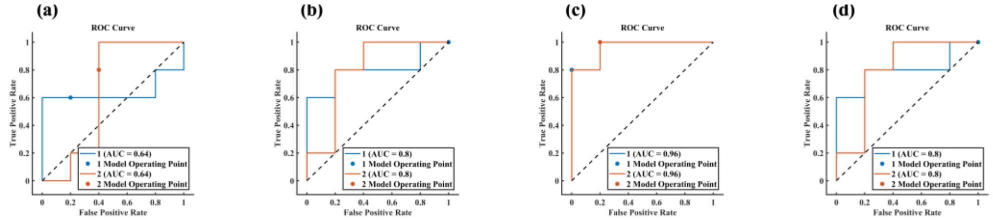

Figure S7. ROC curve of test dataset with (**a**) SVC, (**b**) GS-SVC, (**c**) GA-SVC, and (**d**) PSO-SVC.

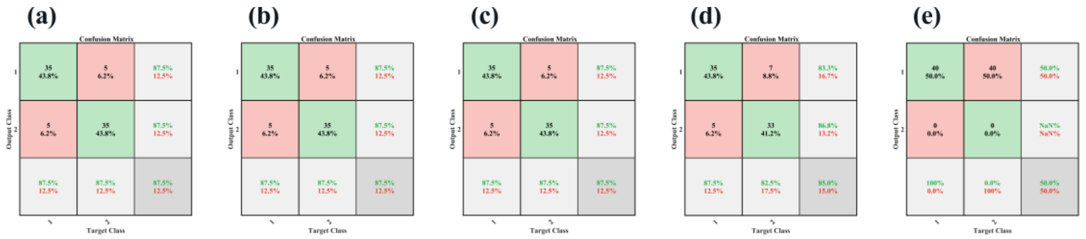

Figure S8. Confusion matrix for the training dataset using nu-SVC with nu values of (**a**) 0.5, (**b**) 0.6, (**c**) 0.7, (**d**) 0.8, and (**e**) 0.9.

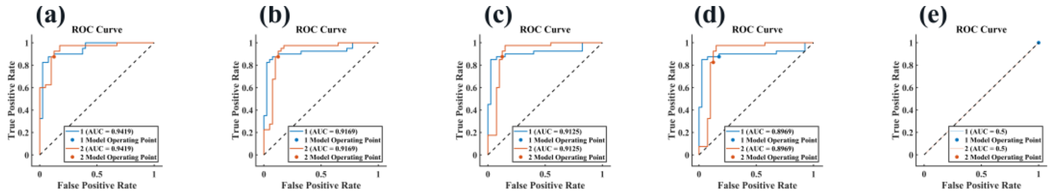

Figure S9. ROC curve for the training dataset using nu-SVC with nu values of (**a**) 0.5, (**b**) 0.6, (**c**) 0.7, (**d**) 0.8, and (**e**) 0.9.

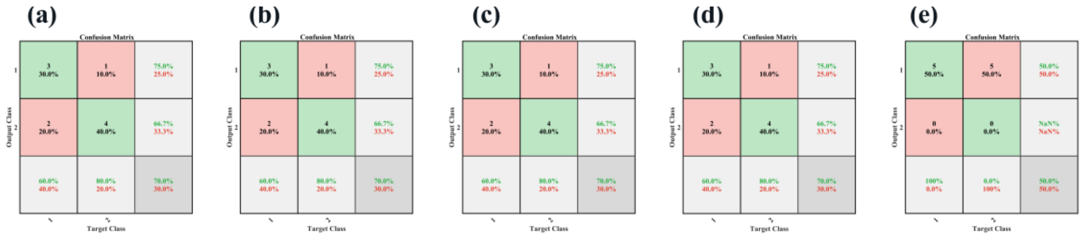

Figure S10. Confusion matrix for the test dataset using nu-SVC with nu values of (**a**) 0.5, (**b**) 0.6, (**c**) 0.7, (**d**) 0.8, and (**e**) 0.9.

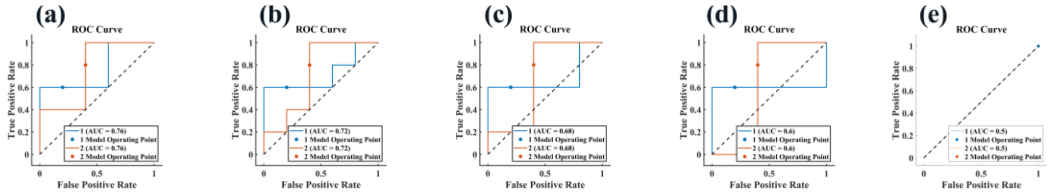

Figure S11. ROC curve for the test dataset using nu-SVC with nu values of (**a**) 0.5, (**b**) 0.6, (**c**) 0.7, (**d**) 0.8, and (**e**) 0.9.

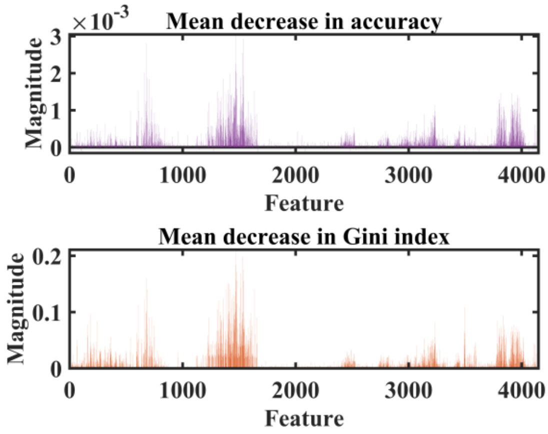

Figure S12. Mean decrease in accuracy and Gini index by RF.

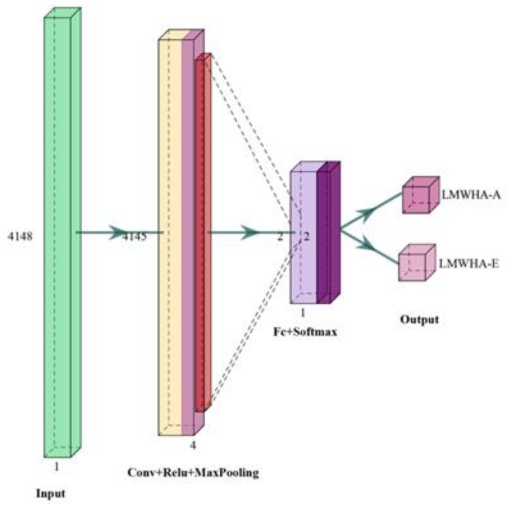

Figure S13. Model architecture of 1D-CNN-7.

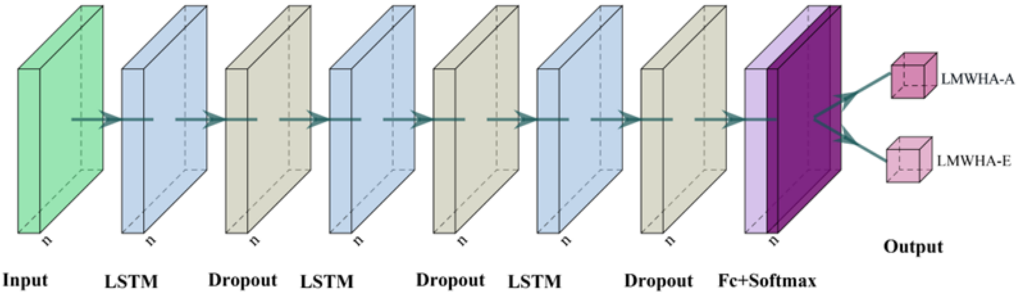

Figure S14. Model architecture of LSTM.

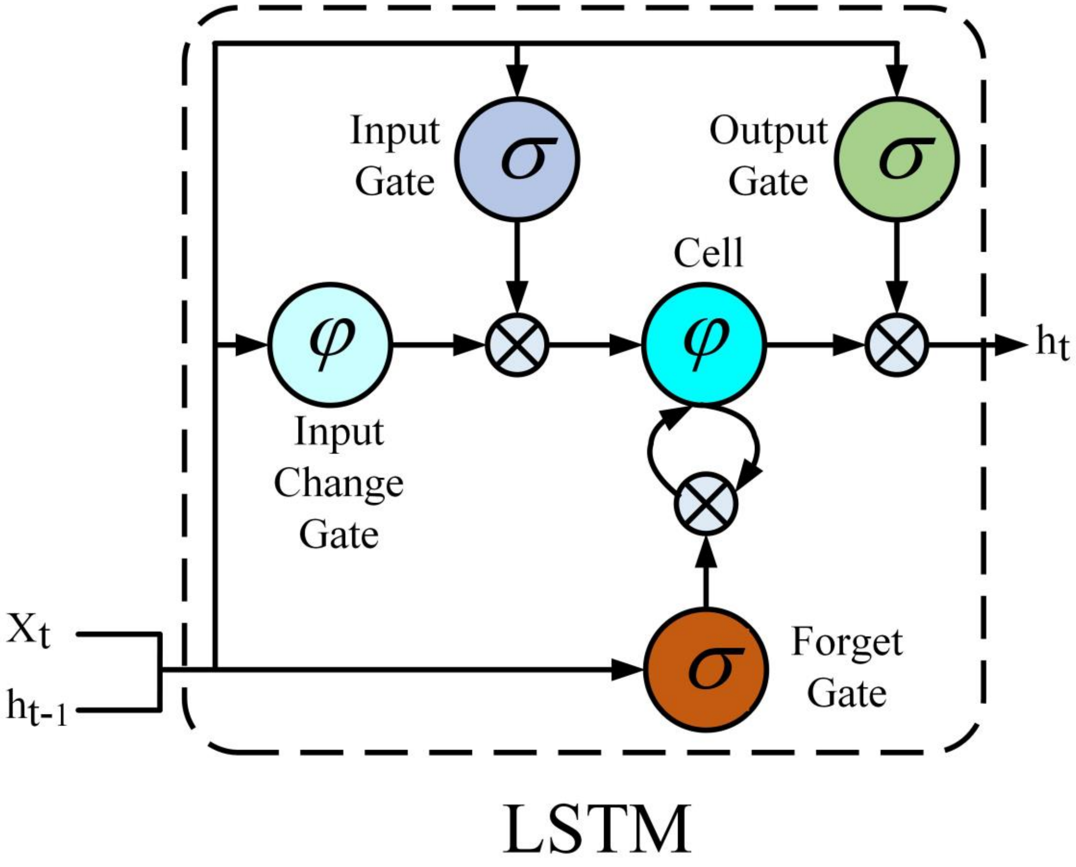

Figure S15. The basic unit of LSTM [65].

$$f_t = \sigma * \left(w_f[h_{t-1}, x_t] + b_t\right) \tag{S1}$$

$$i_t = \sigma * (w_i[h_{t-1}, x_t] + b_i) \tag{S2}$$

$$\tilde{c}_t = \tanh * (w_c[h_{t-1}, x_t] + c) \tag{S3}$$

$$c_t = f_t * c_{t-1} + i_t * \tilde{c}_t \tag{S4}$$

$$o_t = \sigma * (w_0[h_{t-1}, x_t] + b_0) \tag{S5}$$

$$h_t = o_t * \tanh(c_t) \quad \text{(S6)}$$

Where f_t is the forget gate, i_t is the input gate, $\tilde{c}_t$ is the input change gate and calculated for cell state equation, c_t is the cell state, o_t is the output gate, h_t is the hidden layer and b is the bias: σ and tanh represent the activation functions of sigmoid and hyperbolic tangent respectively.

Supplementary Table

Table S1. WAMACS in the area of the first overtone of water (1300–1600 nm) in NIR region [45].

WAMACS	Range (nm)	Wavelength	Attribution
C1	1336-1348	1346	ν_3: H_2O asymmetric stretching vibration
C2	1360-1366	1365	OH-$(H_2O)_{1,2,4}$: water solvation shell
C3	1370-1376	1375	ν_1+ν_3: symmetric and asymmetric stretching vibrations
C4	1380-1390	1387	OH-$(H_2O)_{1,4}$: water solvation shell,<br>O_2-$(H_2O)_4$: hydrated superoxide clusters,<br>ν_1: H_2O symmetrical stretching vibration
C5	1398-1418	1414	S_0: no hydrogen bonds for free water
C6	1420-1428	1426	OH Bend OH...O, hydration band
C7	1343-1444	1440	S_1: water molecules with 1 hydrogen bond (dimer)
C8	1448-1454	1452	OH-$(H_2O)_{4,5}$: water solvation shell
C9	1460-1468	1467	S_2: water molecules with 2 hydrogen bonds (trimer)<br>ν_2+ν_3: H_2O bending and asymmetrical stretching vibration
C10	1472-1482	1478	S_3: water molecules with 3 hydrogen bonds (tetramer)
C11	1482-1495	1491	S_4: water molecules with 4 hydrogen bonds (pentamer)
C12	1506-1516	1513	ν_1: H_2O symmetric stretching,<br>ν_2:H_2O bending vibrations<br>Strongly bound water

C—coordinate; ν—stretching; S—water species.

References

45. Muncan, J.; Tsenkova, R. Aquaphotomics—From innovative knowledge to integrative platform in science and technology. *Molecules* **2019**, *24*, 2742. https://doi.org/10.3390/molecules24152742.

65. Birim, S.; Kazancoglu, I.; Mangla, S.K.; Kahraman, A.; Kazancoglu, Y. The derived demand for advertising expenses and implications on sustainability: A comparative study using deep learning and traditional machine learning methods. *Ann. Oper. Res.* **2022**, *1*, 1-31. https://doi.org/10.1007/s10479-021-04429-x.
